# Demographic and sociocultural risk factors for adulthood weight gain in Hispanic/Latinos: results from the Hispanic Community Health Study / Study of Latinos (HCHS/SOL)

**DOI:** 10.1186/s12889-021-11848-9

**Published:** 2021-11-10

**Authors:** Lindsay Fernández-Rhodes, Nicole M. Butera, Evans K. Lodge, Nora Franceschini, Maria M. Llabre, Elva M. Arredondo, Linda C. Gallo, William Arguelles, Frank J. Penedo, Martha L. Daviglus, Carmen R. Isasi, Paul Smokowski, Penny Gordon-Larsen, Allison E. Aiello, Krista M. Perreira, Daniela Sotres-Alvarez, Kari E. North

**Affiliations:** 1 Department of Biobehavioral Health, College of Health and Human Development, The Pennsylvania State University, University Park, PA Pennsylvania, USA; 2 Carolina Population Center, University of North Carolina at Chapel Hill, NC Chapel Hill, USA; 3 Collaborative Studies Coordinating Center, Department of Biostatistics, Gillings School of Global Public Health, University of North Carolina at Chapel Hill, NC Chapel Hill, USA; 4 Department of Epidemiology, Gillings School of Global Public Health, University of North Carolina at Chapel Hill, NC Chapel Hill, USA; 5 School of Medicine, University of North Carolina at Chapel Hill, NC Chapel Hill, USA; 6 University of Miami, Miami, FL USA; 7 Department of Psychology, San Diego State University, CA San Diego, USA; 8 Baptist Health South Florida, Coral Gables, FL USA; 9 Institute for Minority Health Research, University of Illinois at Chicago, Chicago, IL USA; 10 Albert Einstein College of Medicine, Bronx, NY USA; 11 School of Social Work, University of North Carolina at Chapel Hill, NC Chapel Hill, USA; 12 School of Social Welfare, The University of Kansas, Lawrence, KS USA; 13 Department of Nutrition, Gillings School of Global Public Health, University of North Carolina at Chapel Hill, NC Chapel Hill, USA; 14 Carolina Center for Genome Sciences, University of North Carolina at Chapel Hill, NC Chapel Hill, USA

**Keywords:** Hispanic Americans, Latino health, Weight gain, Adults, Emigration and immigration

## Abstract

**Background:**

United States (US) Hispanic/Latinos experience a disproportionate burden of obesity, which may in part be related to demographic or sociocultural factors, including acculturation to an US diet or inactive lifestyle. Therefore, we sought to describe the association between adulthood weight histories and demographic and sociocultural factors in a large diverse community-based cohort of US Hispanic/Latinos.

**Methods:**

We estimated the effect of several factors on weight gain across adulthood, using multivariable linear mixed models to leverage 38,759 self-reported current body weights and weight histories recalled for 21, 45 and 65 years of age, from 15,203 adults at least 21 years of age at the baseline visit of the Hispanic Community Health Study/Study of Latinos (2008–2011).

**Results:**

The average rate of weight gain was nearly 10 kg per decade in early adulthood, but slowed to < 5 kg a decade among individuals 60+ years of age. Birth cohort, gender, nativity or age at immigration, Hispanic/Latino background, and study site each significantly modified the form of the predicted adulthood weight trajectory. Among immigrants, weight gain during the 5 years post-migration was on average 0.88 kg (95% CI: 0.04, 1.72) greater than the weight gain during the 5 years prior. The rate of weight gain appeared to slow after 15 years post-migration.

**Conclusions:**

Using self-reported and weight history data in a diverse sample of US Hispanic/Latinos, we revealed that both demographic and sociocultural factors were associated with the patterning of adulthood weight gain in this sample. Given the steep rate of weight gain in this population and the fact that many Hispanic/Latinos living in the US immigrated as adults, efforts to promote weight maintenance across the life course, including after immigration, should be a top priority for promoting Hispanic/Latino health and addressing US health disparities more broadly.

**Supplementary Information:**

The online version contains supplementary material available at 10.1186/s12889-021-11848-9.

## Background

Although obesity has been a problem in many Westernized countries like the United States (US) since the 1980s, many low to middle income countries like Mexico and other Latin American nations now rival or lead the US in adult or childhood obesity prevalence [1, 2]. Moreover, studies in the US have shown that racial and ethnic minority populations like Hispanic/Latinos are disproportionality affected by obesity [3]. In 2014 43% of US Hispanic/Latino adults were living with obesity compared to 37% of their non-Hispanic/Latino White peers [4], albeit with variability in obesity across Hispanic/Latino backgrounds (or heritages) [5]. Although limited prospective data exist on the etiology of weight gain in this under-studied population, it is thought that such within group differences may reflect differential exposures to either demographic or sociocultural risk factors (e.g. nativity, age at immigration, acculturative stress, etc.) for weight gain, or differential experiences with obesity-related health conditions later in adulthood [6–8].

Independent of childhood or young adult body mass, adulthood weight gain is a risk factor for all-cause morbidity and mortality later in life [9–11], and is more likely to lead to the deposition of extra weight in the abdomen where fat is most metabolically detrimental [12]. The most common long-term health risks of obesity include cardiovascular-related death, cardiometabolic diseases and several cancers [2, 13]. Hispanic/Latino morbidity and mortality represents an ever-growing share of US deaths and disability-adjusted life-years annually [2]. Therefore, weight maintenance for Hispanic/Latino adults or other US immigrant groups is a key target for public health interventions to combat the obesity epidemic and to mitigate worsening cardiovascular risk factors in this population [14, 15].

Roughly half of Hispanic/Latino adults are born outside of the US and its territories, first arriving to one of the 50 US states or the District of Columbia (DC) as adults, at which point many then go on to begin families in the US [16, 17]. As of 2016, Hispanics/Latinos comprised more than 17.6% of the US population [18]. The importance of studying the health of this diverse and growing US minority group is further emphasized by the fact that between 2016 and 2017, Hispanics/Latinos contributed more than half of the total growth in the US population [19].

In light of the changing nature of US demographic trends [16] and the obesity epidemic in the Western Hemisphere [20], self-reported weight histories such as those collected in Hispanic Community Health Study/Study of Latinos (HCHS/SOL) may offer insights into the origins of, and potential intervention targets for, healthy weight promotion. Herein, we aimed to first summarize the key demographic (e.g. birth cohort, gender, city of residence) and sociocultural characteristics (e.g. Hispanic/Latino background, nativity, age at immigration) associated with adulthood body weight trajectories using baseline data from the diverse, community-based HCHS/SOL (2008–2011). In a second aim, among adult immigrants to the US, we assessed the impact that timing of immigration had on their adulthood weight trajectories, and how this timing of immigration interacted with the same demographic and sociocultural variables considered in the first aim.

## Methods

### Study population

The HCHS/SOL is a community-based cohort of 16,415 adults (18–76 years at baseline examination, 2008–2011) of diverse self-identified Hispanic/Latino backgrounds (Central or South American, Cuban, Dominican, Mexican, Puerto Rican, Other/Multiple) who were living in one of four US urban communities between 2008 and 2011 (Bronx, NY; Chicago, IL; Miami, FL; San Diego, CA, USA) [21, 22]. Based on a participant’s preference, centrally trained bilingual study personnel conducted screening and baseline questionnaires and examinations in either English or Spanish. Women who were pregnant during screening were rescheduled for their baseline examination approximately 3 months after delivery. Manuals of procedures and all administered baseline questions and forms can be found here: https://sites.cscc.unc.edu/hchs/manuals-forms.

### Study design and inclusion criteria

All analyses were restricted to participants > 21 years at the baseline examination (excluded *n* = 1013), and those with no missing demographic or sociocultural covariates (excluded *n* = 106). The resulting complete case analysis yielded a sample size of 15,203 individuals and a total of 38,759 reported weights (from either current age or the weight history). The number of possible observations in the weight history questionnaire was a function of an individual’s current age. For example, individuals < 45 years of age would be able to report a 21 year old and current body weight. Individuals, 45 years of age or older would be able to report up to two (at 21 and 45 years) or three body weights (at 21, 45, and currently). Lastly, individuals aged 65 years or older would be able to report up to three body weights (at 21, 45, 65 years) or four (21, 45, 65 years and currently). In sum, roughly 5 % of the sample reported a weight for one age, 41% reported for two ages, 48% reported for three ages, and 6% reported for four ages. Among the 813 individuals contributing only one body weight, only 84 (10.3%) did not provide a self-reported current weight from the anthropometry questionnaire (i.e. this single weight came from the weight history questionnaire for 21, 45, or 65 years of age).

Separately to address aim 2, we restricted to the subpopulation of 8830 immigrants who came to the US first at > 21 years of age and their 23,518 self-reported weights (with an average of 13.5 years pre-immigration and 15.3 years post-immigration time; and a maximum of 52 years pre-immigration and 52 years post-immigration time). In this sub-sample, roughly 5% reported a weight for one time-point, 32% reported for two time-points, 55% reported a weight for three time-points, and 8% reported a weight for four time-points.

### Self-reported body weights and quality control

As part of the anthropometric questionnaire, all participants (18–76 years) were asked to self-report their current body weight to the nearest lb. or kg. prior to a standarized measurement of body weight [22]. In addition, participants who were at least 21 years old were asked to complete a weight history questionnaire to provide ‘best guesses’ of their non-pregnant body weights (in whole lb. or kg.) for 21, 45, and 65 years of age.

Numerous previous studies have examined both the validity of self-reported weight [23–26] and weight histories [27–36] in various observational and clinical contexts, but most of these analyses have been conducted on smaller samples. Remarkably few studies have included US Hispanic/Latinos [29, 33, 35, 36]. Therefore, below we also discuss HCHS/SOL assessments of reliability and validity.

First, our previous work in HCHS/SOL has demonstrated good reliability in the current self-reported weights from the anthropometry questionnaire (mean difference between original and replicate = 0.46 kg; Coefficients of variation, CV = 6.3%; *n* = 560 participants during same visit) [37]. In addition, we have found good repeatability of the weight reported from the weight history questionnaire (mean difference = − 1.34 to 0.23 kg; CVs = 3.7 to 7.7%; *n* ≤ 52 participants a median of 40 days later) [38], similar to a separate work on the reproducibility of recalled body weight [39]. For purposes of informing the reliability of the self-reported weight history reports made during the same examination, and by participants of varying ages, we also examined the subset of 579 HCHS/SOL participants who were aged exactly 21, 45 or 65 years and who completed both the weight history and anthropometry questionnaires (Supplemental Fig. 1). Self-reported (current) weights from the weight history questionnaire were on average ≤ 0.54 kg less than the anthropometric questionnaire for the same age, and the reliability between these reported weights was generally good (intra-class correlation coefficient ≥ 0.86). Nonetheless the mean differences between self-reported current weights were lowest for individuals at 21 years, followed by 45 years of age, suggesting that self-reports may be least reliable for older adults (e.g. 65 years of age) due to aging or other processes.

Second, we have previously described the accuracy of self-reported current weights relative to measured weight (self-reported versus measured mean difference of 0.23 kg, standard deviation of 4.29); only 4.8% of the observed differences were more extreme than the Bland Altman 95% Limits of Agreement (− 8.18, 8.64 kg) [37]. In fact, 89.3 and 80.2% of the differences were within 5.5 kg of the mean (i.e. a 80% Limit of Agreement: − 5.27, 5.73 kg) or within 5% of the measured weight, respectively. These observations were more accurate (based on any available difference-based metrics) than numerous previous reports on primarily non-Hispanic/Latino samples of smaller size [30–34]. This previous study identified a number of factors associated with mis-reporting of current weight including: age group, gender, body mass index categories, nativity (defined as 50 US states or DC, or elsewhere), a cross-classification between site and background, unit (lb or kg) as well as end digit preference (e.g. 0 s or 5 s, vs. 1–4, 6–9) of self-reported weight. Many of these same factors were of interest as potential correlates of adulthood weight change, and thus were included either as covariates or as effect measure modifiers (e.g. gender, nativity, background by site) in the current study’s statistical analyses. Among the 16,415 HCHS/SOL participants, 16,355 provided at least one self-reported weight. We have previously applied a staged-data quality control protocol that filtered on biologic plausibility, the magnitude of reported weight fluctuations, and body mass index (BMI) of 16–70 kg/m^2^; and excluded reports occurring during pregnancy or made by individuals with a limb amputation [38]. This protocol has yielded a quality-controlled dataset of 39,984 self-reported weights from 16,322 HCHS/SOL individuals for this current study. Lastly, we rounded all weights to the whole kg. to minimize the impact of measurement error [37] based on the unit of self-report (lb. or kg.).

### Statistical analyses

In aim 1, we sought to use all of the available self-reported weight data to model weight trajectories across age using a linear mixed model to describe their interactions with demographic and sociocultural characteristics. The effects for the final linear mixed model for aim 1 (Model 1) are listed in Supplemental Table 1. Weight trajectories were modeled as quadratic in terms of the age for which the weight was reported (‘report age’). In addition, the following covariates were included in the models: age at baseline examination (to account for the amount of elapsed time between the recall and the participant’s current age), birth cohort (before 1980, or 1980 or after; to account for the temporal trends in obesity prevalence), self-identified gender, study site, Hispanic/Latino background, and a cross-classification of nativity (US 50 states/DC, or elsewhere) and age at immigration (first reported arrival to US at 0–11, 12–21, 22–34, 45–44, 45–54, 55–64, 65+ years, with the final categories determined by the availability of data in each model). Based on this coding, individuals born in Puerto Rico were considered to be ‘foreign-born’ and were categorized by the age at which they first moved to one of the 50 US states or DC. Based on previous work in HCHS/SOL [37], we also included an indicator of the self-reported weight end digit preference for zeros and fives as a covariate in the model to minimize the potential for measurement error in the reporting of body weights. The weight trajectory model allowed random intercepts for primary sampling unit, household, and the individual, and a random individual-specific linear slope for report age.

Weight trajectories across age were allowed to differ by birth cohort, gender, place of nativity/age at immigration, Hispanic/Latino background, and study site by including interaction terms with linear and quadratic age terms. We did a joint test for the interactions between the linear and the quadratic terms with report age using a Wald test in the Stata *test* command at an alpha of 0.05. Study site and Hispanic/Latino background were highly collinear in HCHS/SOL, and so study site was found to be interrelated with the association of Hispanic/Latino background with body weight trajectories. Therefore, a variable representing the cross-classification of study site and Hispanic/Latino background (‘background-by-study site’) was constructed and included in all models, where all combinations of Hispanic/Latino background and study site with inadequate sample size (*n* < 100) were pooled into an “other” category. As shown in Table 1, this cross-classification resulted in 13 categories representing a distinct combination of background and study site. To assess whether weight trajectories differed by background, the following nested testing procedure was used. First, we did an overall test for the interaction of linear and quadratic terms for report age with the 13 non-pooled categories of the background-by-study site variable at the 0.05 significance level. If the overall test was statistically significant, then within each study site we further tested the interaction of the linear and quadratic report-age terms with all backgrounds, using a Bonferroni correction for multiple comparisons.

**Table.1:** Descriptive Characteristics^a^ of the Target Population of HCHS/SOL, for the Entire Analytic Sample and the Adult Immigrant Subpopulation^b^

	*Overall* *(Unweighted n = 15,203)*		*Adult Immigrant Subpopulation*^*b*^*(Unweighted n = 8830)*		
*Characteristic*^*a*^	*Unweighted N*	*Weighted % or Mean*	*Unweighted N*	*Weighted % or Mean*	*P-Value*^*c*^
*Male (%)*	6024	47.5 (46.4, 48.6)	3302	45.3 (43.9, 46.8)	0.0001
*Age (%)*					<0.0001
*22–29 years*	1628	18.4 (17.2, 19.6)	212	4.8 (4.1, 5.6)	
*30–39 years*	2360	23.6 (22.2, 24.9)	1155	21.1 (19.5, 22.7)	
*40–49 years*	4176	24.5 (23.5, 25.5)	2571	28.5 (27.0, 29.9)	
*50–59 years*	4286	18.0 (17.1, 18.9)	2834	22.7 (21.4, 23.9)	
*60–69 years*	2254	11.9 (11.1, 12.7)	1660	17.1 (15.9, 18.4)	
*70–76 years*	499	3.6 (3.1, 4.1)	398	5.7 (4.9, 6.6)	
*Study Site (%) Bronx*	3729	28.4 (25.5, 31.5)	1662	20.7 (17.8, 24.0)	<0.0001
*Chicago*	3876	15.8 (13.9, 17.8)	2076	12.7 (10.9, 14.8)	
*Miami*	3872	30.8 (26.6, 35.2)	3141	44.8 (39.3, 50.3)	
*San Diego*	3726	25.0 (21.8, 28.7)	1951	21.8 (18.1, 26.0)	
*Background (%)*					<0.0001
*Central American*	1639	7.6 (6.6, 8.8)	1211	9.3 (8.0, 10.8)	
*Cuban*	2243	21.1 (17.9, 24.7)	1865	32.1 (27.4, 37.1)	
*Dominican*	1332	9.5 (8.2, 10.9)	910	10.2 (8.7, 12.0)	
*Mexican*	5996	36.6 (33.4, 39.9)	3291	32.7 (28.7, 36.9)	
*Puerto Rican*	2554	16.3 (14.8, 18.0)	596	6.5 (5.5, 7.6)	
*South American*	1017	5.1 (4.5, 5.8)	809	7.3 (6.4, 8.3)	
*Other/Multiple*	422	3.8 (3.2, 4.4)	148	2.0 (1.6, 2.5)	
*Background (% within Site) Bronx Dominican*	1245	31.4 (28.2, 34.7)	847	46.0 (41.4, 50.6)	<0.0001
*Central American*	198	5.1 (4.0, 6.3)	127	6.8 (5.2, 8.8)	
*Mexican*	191	10.9 (8.5, 14.0)	115	14.7 (11.0, 19.5)	
*Puerto Rican*	1714	41.7 (38.4, 45.2)	381	20.8 (17.7, 24.2)	
*South American*	180	4.7 (3.7, 5.9)	123	7.8 (6.1, 10.0)	
*Chicago Central American*	406	7.1 (5.8, 8.6)	286	9.9 (7.8, 12.4)	
*Mexican*	2264	61.9 (58.5, 65.1)	1304	68.7 (64.7, 72.4)	
*Puerto Rican*	726	21.0 (17.8, 24.5)	160	8.0 (6.1, 10.5)	
*South American*	351	6.3 (5.0, 7.8)	267	9.7 (7.8, 12.1)	
*Miami Central American*	981	15.6 (12.8, 19.0)	764	14.2 (11.5, 17.4)	
*Cuban*	2171	67.0 (62.3, 71.3)	1824	70.7 (66.1, 74.9)	
*South American*	445	8.3 (6.9, 10.1)	387	9.1 (7.3, 11.1)	
*San Diego Mexican*	3506	93.2 (91.2, 94.8)	1848	93.5 (90.8, 95.4)	
*Born in/after 1980 (%)*	1768	20.0 (18.8, 21.2)	254	5.6 (4.9, 6.5)	<0.0001
*Age at baseline exam (years)*	15,203	43.6 (43.2, 44.1)	8830	48.8 (48.3, 49.3)	<0.0001
*Nativity/age at immigration category (%) US-born*	2255	18.8 (17.4, 20.2)	–	–	–
*0–11 yrs*	1118	8.7 (7.9, 9.6)	–	–	
*12–21 yrs*	3000	20.0 (18.7, 21.3)	–	–	
*22–34 yrs*	4687	29.3 (28.2, 30.5)	4687	55.9 (53.8, 57.9)	
*35–44 yrs*	2313	12.4 (11.5, 13.5)	2313	23.7 (22.3, 25.1)	
*45–74 yrs*	1830	10.7 (9.7, 11.9)	1830	20.4 (18.9, 22.0)	
*Age at immigration (years)*	12,948	22.6 (21.8, 23.4)	8830	35.5 (35.0, 36.0)	<0.0001
*Immigrated in/after 1980 (%)*	10,653	74.3 (72.5, 76.0)	7907	91.6 (90.5, 92.5)	<0.0001
*Time between immigration and baseline exam (years)*	12,948	21.0 (20.3, 21.7)	8830	13.3 (12.8, 13.8)	<0.0001
*Weight at 21 years (kg)*	15,203	65.0 (64.6, 65.5)	8830	61.6 (61.2, 62.0)	<0.0001
*Time between 21 years old and baseline exam (years)*	15,203	22.6 (22.2, 23.1)	8830	27.8 (27.3, 28.3)	<0.0001
*Weight at 45 years (kg)*	9090	72.6 (72.2, 73.1)	6164	71.2 (70.6, 71.7)	<0.0001
*Time between 45 years old and baseline exam (years)*	9090	11.3 (11.0, 11.6)	6164	12.1 (11.7, 12.4)	<0.0001
*Weight at 65 years (kg)*	1228	74.9 (74.0, 75.9)	939	74.2 (73.1, 75.3)	0.0033
*Time between 65 years old and baseline exam (years)*	1228	3.7 (3.5, 4.0)	939	3.9 (3.6, 4.1)	0.0090

^a^ All values (except for N) weighted for study design and non-response

^b^ Adults who migrated to the 50 US states and DC at >21 years old

^c^ P-values came from a test comparing the adult immigrant subpopulation to the subpopulation who did not migrate during adulthood (i.e., who were either born in the US or migrated prior to adulthood). A t-test was used to compare subpopulation means for continuous variables, and a chi-square test was used to compare subpopulation proportions for categorical variables. Due to the inherent differences in the two subpopulations by nativity/age at immigration categories (i.e. non-overlapping categories in the two subpopulations), we did not calculate any statistics for this comparison

In aim 2, we intended to leverage the large adult immigrant subpopulation of HCHS/SOL to describe the weight trajectories across time since immigration and their interactions with demographic and sociocultural characteristics. As shown in Supplemental Table 2, Model 2 contrasted weight trajectories pre/post immigration that were quadratic in terms of time since immigration for each reported weight, and included covariates for age at examination, immigration cohort (before 1980, or 1980 or after; to account for the temporal trends in obesity prevalence upon arrival to US), age at immigration, gender, Hispanic/Latino background, study site, and end digit preference for zeros and fives. This model allowed random intercepts for primary sampling unit, household, and the individual, and a random individual-specific slope for time since immigration. Again, weight trajectories across time since immigration were allowed to differ by immigration cohort, gender, age at immigration, and Hispanic/Latino background by study site. The pre-immigration trajectory was allowed to differ from the post-immigration trajectory, and this difference in the shape of the pre-immigration and post-immigration trajectories was tested at an alpha of 0.05. Specifically, we included (1) two-way interaction terms between time since immigration (linear and quadratic terms) and the demographic and sociocultural characteristics mentioned above, and (2) three-way interaction terms between time since immigration (linear and quadratic terms), demographic and sociocultural characteristics, and pre/post immigration. Similar to above, the weighted mean height for the adult immigrant subpopulation, or their corresponding stratum-specific weighted mean heights, were used to calculate the weight and time point at which an average height individual would first be at a body mass index ≥30 kg/m^2^ and was signified with triangles in the figures.

Lastly as part of a sensitivity analysis, we conducted the same modeling as described above for Models 1 and 2, but restricted our analytic sample to the subpopulation of HCHS/SOL who reported weights consistent with having a BMI < 30 kg/m^2^ at 21 years of age. Given that we had already considered an individual’s 21 year old weight in the creation of this subpopulation, we only analyzed (Model 1 subsample: *n* = 13,125; Model 2 subsample: *n* = 7763) and estimated weight trajectories for those who reported at least one other weight between 22 and 74 years of age.

All linear mixed models accounted for the complex sampling design by including random intercepts for primary sampling units, household, and individual, and applying sampling weights to the level of the model corresponding to the individual. All linear mixed models were estimated using the *mixed* command in Stata 14 using an independent correlation matrix for all random effects and independent residuals (StataCorp LP, College Station, TX, USA). Trajectories were plotted based on the covariate distribution of the analytic sample, unless otherwise noted in the footnote of each main and supplemental figure. Therefore, within a given panel the plotted differences in trajectories should due to the specific demographic or sociocultural strata that are being plotted. The overall weighted mean height in our HCHS/SOL analytic sample, or in the case of trajectories by demographic/sociocultural characteristics the stratum-specific weighted mean heights, were used to calculate the weight and age at which an average height individual would first be at a body mass index ≥30 kg/m^2^. Triangles were placed on the resulting figures to indicate this transition point(s).

## Results

Table 1 describes the characteristics among all adults, as well as the immigrant subpopulation who first arrived to the 50 US states/DC at > 21 years of age (*n* = 8830). Differences were seen between the adult immigrant subpopulation versus all others across characteristics (*p* ≤ 0.009). For example, the adult subpopulation appeared to have more females and be older than the overall population. Weighted frequencies and means revealed that the target population of HCHS/SOL was mainly Mexican American, followed by Cuban and Puerto Rican. As compared to the overall population, the adult immigrant subpopulation included proportionally more Cubans and less Puerto Ricans. The majority of the overall population, and adult immigrant subpopulation, was born before 1980, and first immigrated to the US in 1980 or after—most often between the ages of 22–34 years. In the entire target population, this was 21 years prior to the baseline examination on average, and among the adult immigrant sub-population this was 13 years prior to baseline. Lastly, the amount of time that participants were asked to recall back varied as a function of their own age at baseline and the requested age of report, and was greatest on average for 21-year-old reports (weighted mean of 22.6 years past) and least for 65-year-old reports (3.7 years). Nonetheless, the majority of the self-reported weight data (57.9%, unweighted frequency) was reported within 10 years of the participant’s current age.

**Fig. 1:**
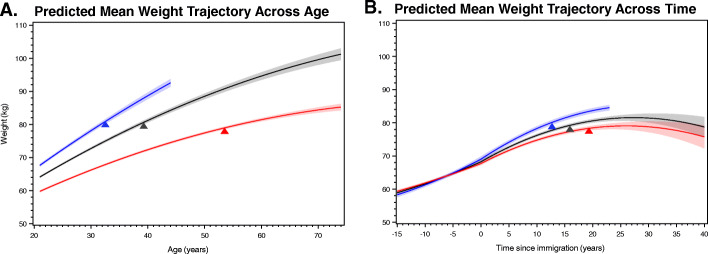
**A**-**B** Predicted Weight Trajectories Across Age (**A**) and Time since Immigration (**B**) for All Hispanic/Latino Adults from the Hispanic Community Health Study/Study of Latinos (HCHS/SOL) Baseline Examination (2008–2011), and for Those Individuals <45 Years Old (Blue) or ≥45 Years (Red) Old at Baseline. **A** The gray weight trajectory and 95% Confidence Interval (CI) reflects the weighted average age at examination (43.6 years), proportion male (47.5%), proportion born before 1980 (80.0%), nativity/age at immigration categories, proportion for each background by study site combination (constructed to represent combinations of more than ≥100 individuals, see Table 1), and proportion with digit preference for self-reports ending in 0s or 5s (79.5%). **B** Among individuals who immigrated to the US first as adults (>21 years), the gray weight trajectory and 95% CI reflects the weighted average at examination (48.8 years), proportion male (45.3%), proportion immigrating before 1980 (8.4%), average age at immigration (35.5 years), proportion for each background by study site combination (see Table 1), and proportion with digit preference for self-reports ending in 0s or 5s (75.7%). The colored weight trajectories are based on the same model coefficients, but reflect the average adjustments and range of observed time points for the subset of participants who were either <45 years (blue) or ≥45 years (red) at baseline. The test of difference in pre/post immigration slope was significant (Chi-square *p*-value<0.0001). Examples of how to calculate population-level weight change, or the effect of demographic or sociocultural factors, based on the final model coefficients are provided as part of Supplemental Tables 3–4

The weight trajectory analysis suggested that independent of covariates (see model coefficients and example calculation in Supplemental Table 3), adults in this cohort gained weight at an average rate of 9.5 kg per decade (95% CI: 6.8, 12.3; Table 2) between 22 to 29 years of age, but this rate appeared to slow across time (Fig. 1A). The average rate of weight gain between 30 and 39 years of age was nearly 8.5 kg per decade (95% CI: 6.2, 10.7), and continued to slow to 7.3 kg per decade (95% CI: 5.0, 9.6) by mid-adulthood (i.e. 40s), and to 5.0 kg per decade (95% CI: 2.6, 7.3) by 60 to 69 years of age (Table 2).

**Table.2:** Average Weight Gain per Age Range^a^ and 95% Confidence Intervals (CIs) Based on Mixed Effects Modeling

*Age Range*	*Average Weight Gain Per Age Range*^*a*^	*Lower 95% CI Limit*	*Upper 95% CI Limit*
22–29 years	9.52634	6.76798	12.2847
30–39 years	8.47104	6.24663	10.6955
40–49 years	7.29849	5.04409	9.5529
50–59 years	6.12593	3.83046	8.4214
60–69 years	4.95338	2.60634	7.3004
70–76 years	4.01533	0.16987	7.8608

^a^ Average weight gain per decade of age is calculated as the fitted value from the model for the end of the age range minus the fitted value for the beginning of the age range, standardized to 10 years (i.e., divided by the length of the age range, and then multiplied by 10 years). The fitted values were adjusted to weighted age at clinic visit, proportion male, proportion born before 1980, distribution of age at immigration, proportion for each site-background combination, and proportion preferring 5 s and 10s

Figure 1B shows the weight trajectory among the 8830 adult immigrants by time since first migration to the US. As described above, we allowed the shape of the weight trajectory to differ before and after immigration by including interactions for the linear and quadratic terms for time since immigration with an indicator for post-immigration. We tested whether the shape of the trajectory differed during the pre/post-immigration periods (i.e. before and after the zero in the x-axis, see Supplemental Table 4 for an example). After accounting for covariates, there was an average weight gain of 3.5 kg in the five years before immigration, in contrast to 4.4 kg in the five years after immigration, resulting in an increase of 0.88 kg (95% CI: 0.04, 1.72) weight gain across this combined 10 year period. Yet, the rate of weight gain appeared to begin to slow after 15 years post-migration (Fig. 1B). This slowing post-immigration weight trajectory resulted in an apparent leveling off of weight gain between 15 and 40 years post immigration.

**Fig. 2:**
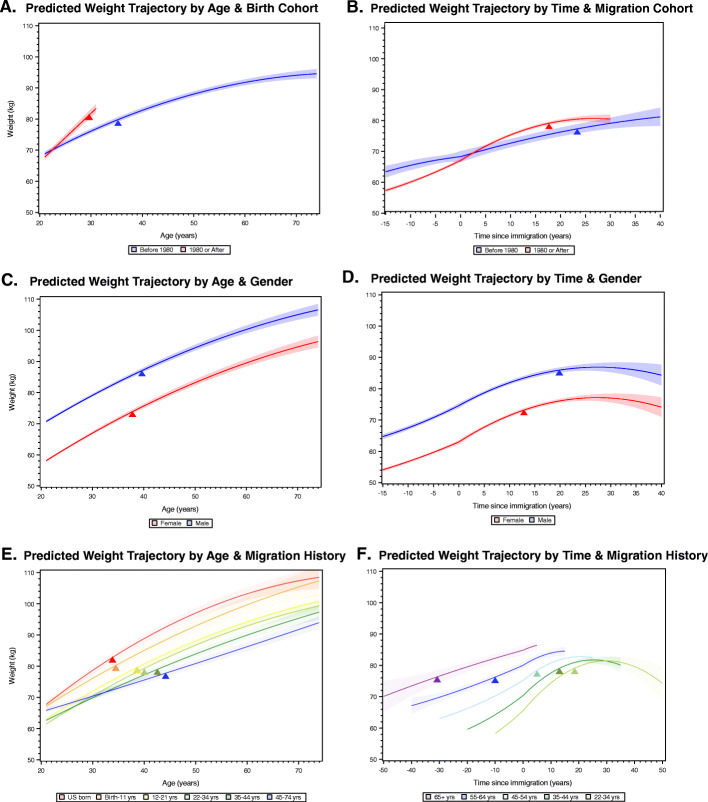
**A**-**F** Modifiers of Predicted Weight Trajectories Across Age (**A**, **C**, **E**) and Time since Immigration (**B**, **D**, **F**) for Hispanic/Latino Adults from the Hispanic Community Health Study/Study of Latinos (HCHS/SOL) Baseline Examination (2008–2011). Mean weight trajectories and 95% Confidence Intervals (CIs) across age shown in **A**, **C** and **E** represent adjustments for the following weighted covariates or interactions with their displayed categorizations: age at examination (except for **A** that plots the average for a 30 year old), proportion male, born before 1980, nativity by age at immigration combination, background by study site combination (constructed to represent combinations of more than ≥100 individuals), and digit preference for self-reports ending in 0s or 5s. Mean weight trajectories and 95% CIs across time since immigration in **B**, **D** and **F** represent adjustments for the following weighted covariates or interactions with their displayed categorizations: age at examination (except for **B** that plots the average for a 55 year old), proportion male, immigration before 1980, age at immigration, background by study site, and digit preference for self-reports ending in 0s or 5s. Pre/post immigration weight trajectories varied by the categorical variables shown in **B**, **D**, and **F** (Chi-square p-values being <0.0001 to 0.01). In addition, **F** show the average trajectory for the mid-point of each age at immigration category. The Chi-square test of difference in the pre/post immigration slopes was significant in all cases in **B**, **D** and **F** (*p*<0.0001). The tips of the colored triangles in all panels signify the point (if any) in the weight trajectory where the average body mass index rises above 30kg/m^2^, given the weighted mean height of that particular subgroup. Examples of how to calculate population-level weight change, or the effect of demographic or sociocultural factors, based on the final model coefficients are provided as part of Supplemental Tables 3–4

**Fig. 3:**
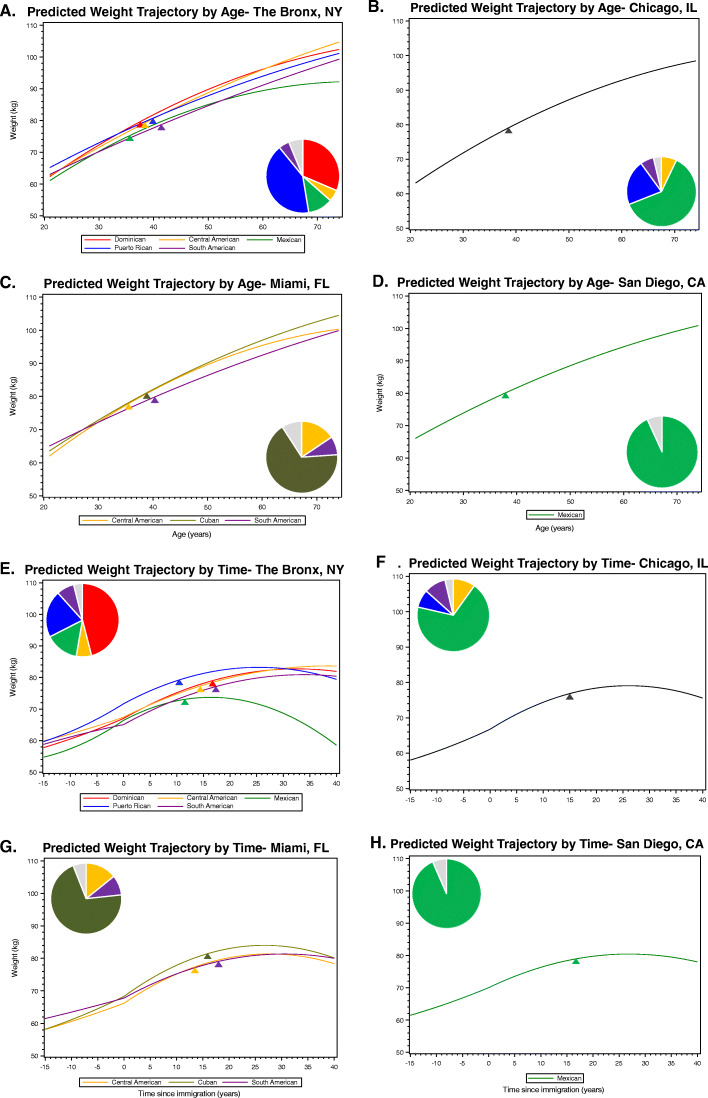
**A**-**H** Hispanic/Latino Background Differences in Predicted Weight Trajectories and Weighted Frequencies by Study Site and across Age (**A**-**D**) and Time since Immigration (**E**-**G**) for Hispanic/Latino Adults from the Hispanic Community Health Study/Study of Latinos (HCHS/SOL) Baseline Examination (2008–2011). All weight trajectories and pie chart weighted frequencies for Central Americans are shown in gold, Cubans in dark green, Dominicans in red, Mexicans in light green, Puerto Ricans in blue, and South Americans in purple, provided that each study site surveyed ≥100 individuals of each background; therefore, some background-site combinations with <100 individuals were not be displayed on each trajectory panel and were instead combined with all other less frequent background-site combinations (shown in light gray in the pie charts). Mean weight trajectories by background group-study site combination (constructed to represent combinations of more than ≥100 individuals) an across age shown in **A**-**D** represent the weighted average age at examination, proportion male, nativity/age at immigration categories, proportion born before 1980, and proportion with digit preference for self-reports ending in 0s or 5s. Within Chicago, background differences did not appear after accounting for multiple comparisons and are shown in a condensed manner (shown in dark gray in **B** and **F**, Chi-square *p*-value=0.025). Mean weight trajectories shown in **E**-**G** represent the weighted average at examination, proportion male, average age at immigration, proportion immigrating before 1980, and proportion with digit preference for self-reports ending in 0s or 5s. Pre/post immigration weight trajectories varied by background within each site (Chi-square *p*-value<0.0001). The Chi-square test of difference in the pre/post immigration slopes was significant in all cases (*p*<0.0001). The tips of the colored triangles signify the point in the weight trajectory where the average body mass index rises above 30 kg/m^2^, given the weighted mean height of that particular subgroup. Examples of how to calculate population-level weight change, or the effect of demographic or sociocultural factors, based on the final model coefficients are provided as part of Supplemental Tables 3–4

Based on the weighted mean height per subgroup, we estimated the age at which individuals from the HCHS/SOL communities/subgroups would begin living with obesity and the estimates are overlaid on Figs. 1, 2, 3 and provided in Supplemental Table 5. Although this transition occurred for the overall trajectory between 38 and 39 years of age, for those adults who were < 45 years of age at the time of the HCHS/SOL baseline examination, this turning point was expected to occur even earlier—in their 30s. Whereas for those 45 years of age or older at baseline, this was expected to happen in their 50s. Among the adult immigrants, the apparent slowing of weight gain ~ 15 years post-migration also appeared around the same time when an average adult immigrant would have been expected to develop obesity (Supplemental Table 5; Fig. 1B).

Adjusted weight trajectories in adulthood varied across a number of demographic and sociocultural risk factors (Fig. 2A-E). For example as shown in Fig. 2A, individuals born in or after 1980 appeared to have a greater average weight gain in their 20s compared to individuals born before 1980. This cohort difference represents an estimated 5.7 ± 1.13 kg of extra body weight by the age of 30 years. Further, in Fig. 2B, we observe a greater post-immigration trajectory of average weight gain and earlier estimated time at which these adult immigrants on average are expected to develop obesity (i.e. by 21 years post immigration versus 27 years for those who came to the US before 1980).

As shown in Fig. 2C-D, gender shifted the estimated intercept (i.e. at 21 years of age) for men upwards by approximately 12.6 kg (SE: 0.33) as compared to women in Fig. 2C and 11.6 kg (SE: 0.47) in Fig. 2D. The form of the trajectories across age and time since immigration varied less dramatically. When taking the weighted average height differences between the genders into account, females were predicted to acquire obesity before their male counterparts; however, this was more dramatic for estimated trajectories across time since immigration than for age, which indicates that female adult immigrants to the US may experience greater immigration-related weight gain than men on average (Fig. 2D).

Nativity and age at immigration were also associated with age and immigration-related weight trajectories (Fig. 2E-F). For example as shown in Fig. 2E, being either born in the US or brought to the US as a child (0–11 years), during adolescence, or young adulthood (12–21 years) resulted in greater age-related weight gain and an earlier average age of obesity onset—between 34 and 38 years of age. Fig. 2E shows that the intercept of the estimated weight trajectories for most adult immigrant groups were shifted downwards, and this, in part, resulted in an older average age of obesity onset, in their 40s instead of their 30s. Fig. 2F shows a similar trend through the perspective of time since immigration; a similar dose-response pattern appeared wherein immigrants, who arrived to the US in early or mid-adulthood (22–44 years), arrived prior to developing obesity. Yet, their this potential advantage appeared to erode with time spent in the US as their weight trajectories converged within 20 years to those of mid-adult immigrants (45–54 years). In contrast, adult immigrants who arrived to the US first in later adulthood, e.g. 55 years or older, on average were living with obesity prior to immigration, which may reflect age-related trends in obesity in Latin America or other correlates of an individual immigrating in late adulthood to the US (e.g. family reunification, accumulated wealth, retirement/employment status, and health status, etc.).

Trajectories of weight gain in adulthood differed by both HCHS/SOL study site and self-identified Hispanic/Latino background (Fig. 3A-H). Because study site was found to be related to Hispanic/Latino background and body weight trajectories, our weight trajectories are presented for each background within each study site. The test for differences in the weight trajectories by background was not significant within the Chicago study site, and so the average weight trajectory across age or time since immigration in the Chicago study site is pooled across backgrounds (Fig. 3B and F). Most background-site groupings gained body weight well into their 70s and the trajectories by background overlapped substantially within each site (Fig. 3A-H).

Based on our modeling most HCHS/SOL participants are expected to begin living with obesity by their mid-30s to mid-40s (Fig. 3A-D). However, adulthood weight gain slowed more after 60 years of age among Mexican Americans and their overall trajectory was statistically significant as compared to Dominicans living in the Bronx (*p*-value = 0.009) (Fig. 3A). More favorable weight trajectories (i.e. earlier leveling off of weight gain) were seen for Central Americans as compared to Cubans (p-value = 0.02) and South Americans (p-value< 0.0001) who were also living in Miami (Fig. 3C). Yet, given that both Mexican Americans in the Bronx and Central Americans in Miami experienced obesity at an earlier onset than the other background groups at the same site, this slowing of weight gain in late adulthood may in part be related to the duration of obesity and its cardiometabolic sequelae, or survival biases.

Our time since immigration analysis (Fig. 3E-H) showed that most background and study site groups being living with obesity 10–20 years after immigration and that this onset was earliest for Puerto Ricans living in the Bronx and Central Americans living in Miami, and latest for South Americans living in both locations (Fig. 3E and G). The estimated pre/post immigration trajectory for the Mexicans living in the Bronx was statistically different compared to all other background groups also living in the Bronx (*p*-values≤0.0056). This implies that not all Hispanic/Latino adults are at equal risk for weight gain or early adulthood onset obesity, and that future studies should investigate further which Hispanic/Latino backgrounds and locations are at greatest risk of cardiometabolic disease and immigration-related stress and how this may be associated with changes in adult body weight.

Lastly as a sensitivity analysis, we plotted the population-level trajectories between 22 and 74 years of age within the subgroup of HCHS/SOL (*n* = 13,125; 59% whom were adult immigrants) who reported body weights consistent with having a BMI < 30 kg/m^2^ at 21 years of age. Although Fig. 1A-B and Supplemental Figs. 2A-B are not directly comparable, we observed substantively similar population-level trends in weight gain (Supplemental Fig. 2A-B). We noted that even though the non-obese subgroup started at a lower weight in early adulthood, they continued to gain weight throughout adulthood, ending up at a similar estimated weight by 70 years of age (~ 100 kg), as what was seen in Fig. 1A-B for the overall sample. This sensitivity analysis indicated that the relatively small group of individuals who were living with obesity at 21 years of age did not appear to be driving our overall findings.

## Discussion

Hispanic/Latino adults comprise the largest racial/ethnic minority group in the US [18] and face a higher prevalence of overweight and obesity than their non-Hispanic/Latino peers [4]. Thus, understanding of the correlates and magnitude of adult weight gain in this population is critical. This is particularly challenging for Hispanic/Latino immigrants, who may be difficult to follow using traditional longitudinal studies and who often receive medical care across borders, from non-traditional providers or go periods of time without health services or insurance [5]. Based on the estimated weight trajectories, the average adult, from one of the four communities studied as part of HCHS/SOL, began living with obesity (≥30 kg/m^2^) before their 40s; however, we observe notable heterogeneity in individual trajectories and timing of obesity onset. Despite the potential for self-report bias, we demonstrate how repeated measures/reports of body weight can help provide insights into the correlates of weight gain, in under-studied or marginalized populations, like US Hispanic/Latinos.

Past studies of US Hispanics/Latinos have noted substantial heterogeneity in obesity burden across Hispanic/Latino backgrounds [6]. Others have described the cross-sectional association between obesity prevalence and sociocultural measures, including proxy measures of acculturation—the dynamic process of adaptation to aspects of a new culture and its associated lifestyle and dietary habits [6, 40, 41]. This body of cross-sectional evidence has supported the ‘unhealthy assimilation’ hypothesis that we would expect immigrant body weights to ‘converge’ to levels seen among US-born Hispanics/Latinos with increasing acculturation to obesogenic US lifestyles. Emerging results from repeated cross-sectional and longitudinal investigations of this hypothesis are mixed, however, indicating that obesity-protective exposures in early and middle life of some immigrants persist across the life course and may lead to ‘divergent’ weight trajectories between foreign and US-born adults [40, 42–45].

In this current study of diverse Hispanic/Latino adults, we focused on describing the form of the relationship between body weight and the demographic and sociocultural factors, which we expected would be relatively constant across time. We observed strong evidence for poor weight maintenance across a wide range of adulthood (21–76 years of age), which is supported by documented epidemiologic trends in this population [3, 4]. By leveraging repeated measures of body weight—occurring both prior to and after immigration—we were able to link the timing of one’s first immigration to the US with increases in weight gain. We found that, while HCHS/SOL participants did experience a statistically significant acceleration in weight gain during the first five years post-immigration, albeit modestly by < 1 kg. The overall predicted trajectory leveled off and therefore did not support ‘convergence’ with US-born Hispanics/Latinos similar to previous findings [40, 42–45]. Furthermore, post-immigration weight gain was buffered by being male, arriving to the US before 1980, or arriving to the US in mid/late adulthood, when one’s lifestyle may be less permeable to obesogenic environments. Although the overall acceleration of weight gain post-immigration was modest (< 1 kg), it may have greater public health significance when considered in conjunction with other obesogenic factors. For example, female adult immigrants developed obesity on ~ 8 years before their male peers (Fig. 2B), or select backgrounds were estimated to develop obesity 5 or more years earlier than other backgrounds living in the same community (Fig. 3E-G).

Even though individuals born in 1980 or after were younger at baseline on average, they may have grown up amidst the US obesity epidemic and had greater exposure to obesogenic environments earlier in life. The trajectory of early adulthood weight gain observed for those born in 1980 or after is particularly concerning as it indicates that this group may experience an earlier transition to obesity—estimated on average to occur in their earlier 30s. Although previous scholarship has studied the association between weight trajectories and a number of risk factors [42, 43, 45–48], to our knowledge this is the first work to describe these weight trajectories in such a large and diverse sample of Hispanic/Latinos across such a wide time span in adulthood, and across borders.

Our finding that immigrant women in the HCHS/SOL on average began living with obesity earlier after arriving to the US as compared to their male peers may point to unique stressors and vulnerabilities faced by immigrant women in the US. Differences in expectations regarding work, remittances, childcare, or experiences of immigration-related trauma may underlie this gender disparity. We also uncovered important differences in adult weight trajectories across the HCHS/SOL study sites and the diverse background groups living there. Such individual and community-level differences deserve additional investigation to fully understand how demographic and sociocultural characteristics may interact to exacerbate or mitigate adult weight gain in this diverse US populations.

There are important limitations of this work to discuss. First, given that we rely on repeated measures of self-reported weights, recall bias may shape our data, increasing either as a function of time that is recalled or with less socially desirable weight statuses [33]. We have previously reported good accuracy (validity) of self-reported weights in the HCHS/SOL, and we consider many of these predictors of misreporting in our modeling [37]. Given the relative dearth of studies in Hispanic/Latino or immigrants populations, we are unsure the exact nature of recall bias relates to these same factors, or if this bias scales linearly with time since recall, as suggested previously [33]. Herein, we adjusted for age at examination as a linear indicator of the time between the reported weight and the baseline observations in our linear mixed modeling.

Furthermore, weight change itself may alter one’s ability to accurately recall weight [31, 32, 34], but this differential bias has not been observed consistently in all US-based nationally-representative samples [29, 33] or for recalled body weights within 10 years [30]. We note that the majority of the self-reported weight data in HCHS/SOL was reported within 10 years of the participant’s current age. Although we may not have the data to directly validate our recalled weights during a time when the majority of our Hispanic/Latino adult participants were preparing/immigrating to the US, we would expect that if self-report and recall biases were to be present, it would most likely result in an attenuation of the estimated weight trajectories by encouraging more moderate reporting of extreme weight values. This assumption is supported by previous observations of differential mis-reporting towards more socially desirable norms in validity studies of non-US populations [32, 34]. Nonetheless, we cannot rule out the role of residual measurement error in the body weights used in this current study.

Due to the complex structure of our data collection, the majority of the weight measurements are recalled at 21, 45, or 65 years of age with the rest being self-reported for a participants current age, which could have ranged from 22 to 76 years. Therefore, there are more early- versus late-adulthood weights, and depending on when immigration occurred in adulthood, we may have had an unbalanced number of observations during the pre versus post-immigration periods. We also cannot rule out the role of selection or survival biases in shaping the type of community-dwelling individuals living within the HCHS/SOL communities and thus the weight trajectories described herein. Lastly, overweight and obese low-income Hispanic mothers from Houston, Texas who reported poor health, were more likely to accurately categorize their body mass than their peers of better self-rated health [49]. This current study did not have access to pre-baseline measures (e.g. health care access, diet quality, physical activity, stress, socioeconomic factors), which could drive changes in health behaviors, ethnic identity, acculturative processes, migration-related/psychosocial stressors or residential mobility, in order to further explore their impact on weight trajectories.

## Conclusions

In this large community-based study of diverse Hispanic/Latino adults, on average individuals reported weights that correspond to a substantial amount of adulthood weight gain. Participant gender was associated with differences in the estimated weight intercept in early adulthood, and greater average weight gain for women post-migration. Several other socio-demographic factors patterned differences in early to mid-adulthood (e.g. birth and immigration cohort, nativity/age at immigration, study site by Hispanic/Latino background). Despite its limitations, this work represents an important first step towards understanding the risk factors for age and migration-related weight gain in this population, and may inform windows of intervention for future studies or initiatives to promote health in US Hispanic/Latino communities.

## Supplementary Information

**Additional file 1.** Supplementary Information.

## Data Availability

The Hispanic Community Health Study/Study of Latinos (HCHS/SOL) data used in this manuscript are publicly available following an approved manuscript proposal. The investigators’ website can be found here. http://www.cscc.unc.edu/hchs/

## Abbreviations

CI: Confidence Interval
BMI: Body mass index
DC: District of Columbia
HCHS/SOL: Hispanic Community Health Study/Study of Latinos
US: United States
