# Supplementary material for: Demographic and sociocultural risk factors for adulthood weight gain in Hispanic/Latinos: results from the Hispanic Community Health Study / Study of Latinos (HCHS/SOL)
Source: BMC Public Health. 2021 Nov 10;21:2064. doi: 10.1186/s12889-021-11848-9 (PMC8582171; doi:10.1186/s12889-021-11848-9)
Supplement: Supplementary file 1 — Additional file 1. Supplementary Information. [file 12889_2021_11848_MOESM1_ESM.docx]

TITLE PAGE

Manuscript Title: Demographic and Sociocultural Risk Factors for Adulthood Weight Gain in Hispanic/Latinos: Results from the Hispanic Community Health Study / Study of Latinos (HCHS/SOL)

**Authors’ Full Names and Affiliations:** Lindsay Fernández-Rhodes^1,2*^, Nicole Butera^3^, Evans K. Lodge^4,5^, Nora Franceschini^4^, Maria M. Llabre^6^, Elva M. Arredondo^7^, Linda C. Gallo^7^, William Arguelles^6,8^, Frank J. Penedo^6^, Martha L. Daviglus^9^, Carmen R. Isasi^10^, Paul Smokowski^11,12^, Penny Gordon-Larsen^2,13^, Allison E. Aiello^2,4^, Krista Perreira^2,5^, Daniela Sotres-Alvarez^3^ and Kari E. North^4,14^

^1^Department of Biobehavioral Health, The Pennsylvania State University, University Park, PA, USA;

^2^Carolina Population Center, University of North Carolina at Chapel Hill, NC, USA;

^3^Collaborative Studies Coordinating Center, Department of Biostatistics, University of North Carolina at Chapel Hill, NC, USA;

^4^Department of Epidemiology, University of North Carolina at Chapel Hill, NC, USA;

^5^School of Medicine, University of North Carolina at Chapel Hill, NC, USA;

^6^University of Miami, Miami, FL, USA;

^7^San Diego State University, San Diego, CA, USA;

^8^Baptist Health South Florida, Coral Gables, FL, USA;

^9^Institute for Minority Health Research, University of Illinois at Chicago, Chicago, IL, USA;

^10^Albert Einstein College of Medicine, Bronx, NY, USA;

^11^School of Social Work, University of North Carolina at Chapel Hill, NC, USA;

^12^School of Social Welfare, The University of Kansas, Lawrence, KS, USA;

^13^Department of Nutrition, University of North Carolina at Chapel Hill, NC, USA;

^14^Carolina Center for Genome Sciences, University of North Carolina at Chapel Hill, NC, USA.

**Corresponding Author’s Name and Contact Information:** Lindsay Fernández-Rhodes, 219 Biobehavioral Health Building, University Park, PA 16802, 814-863-0185, fernandez-rhodes@psu.edu

**SUPPLEMENTARY INFORMATION**


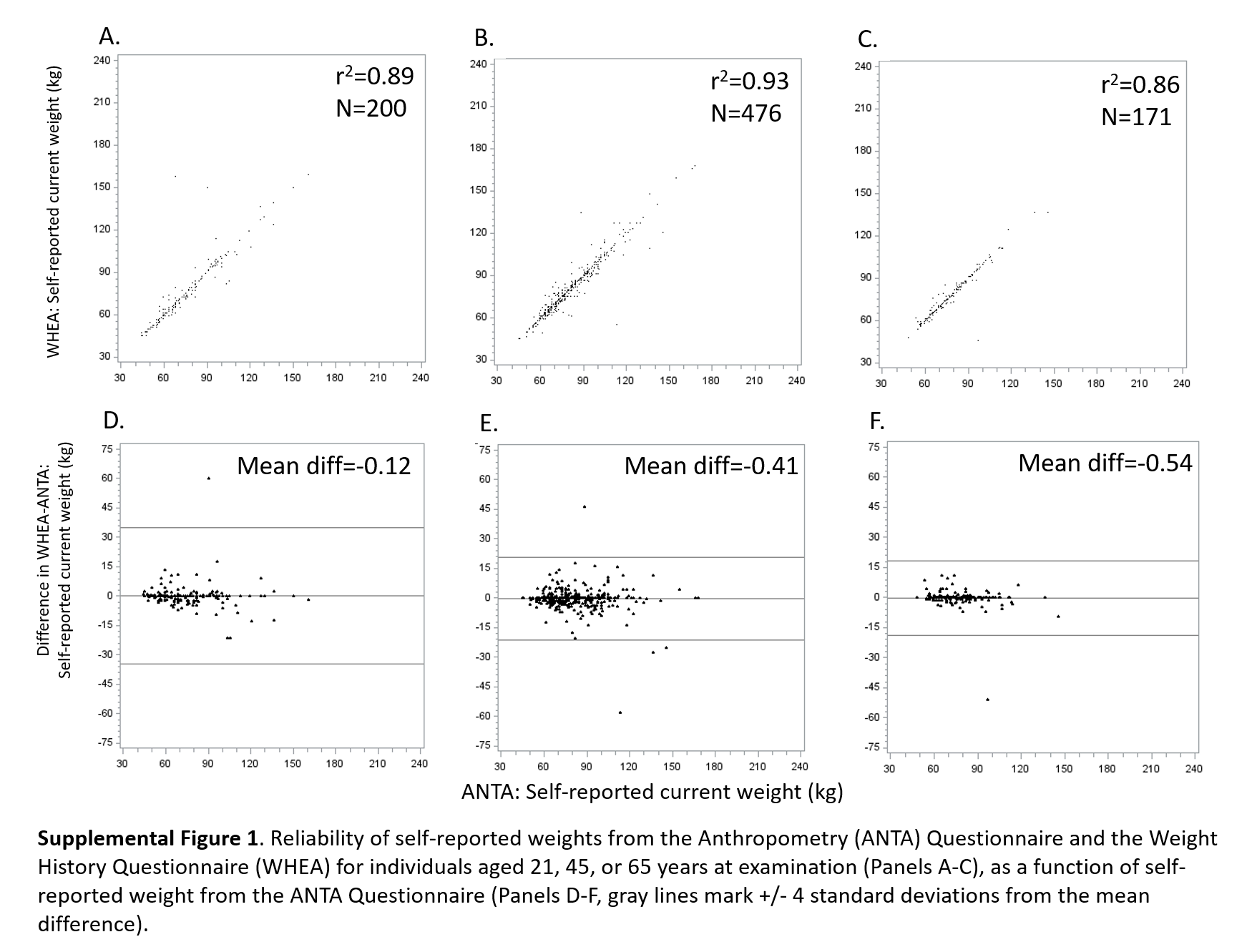

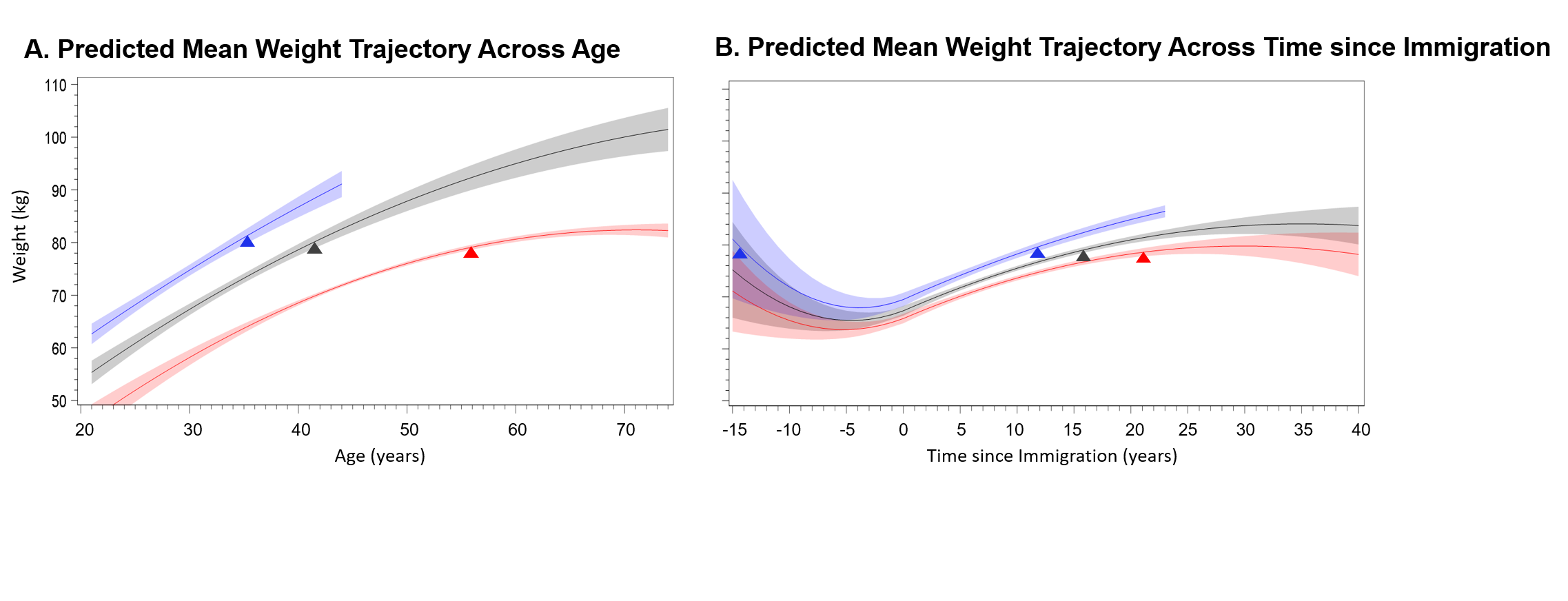


**Supplemental Figure 2A-B.** **Predicted Weight Trajectories Plotted Across Age (Panel A, N=13,125) and Time since Immigration (Panel B, N=7,763) for the Subpopulation That were Not Living with Obesity at Age 21 Years Based on the Hispanic Community Health Study/Study of Latinos (HCHS/SOL) Baseline Examination Data (2008-2011), and Shown Overall (Gray) and Separately for Those Individuals <45 Years Old (Blue) or ≥45 Years (Red) Old at Baseline**

**Panel A.** The gray weight trajectory and 95% Confidence Interval (CI) reflects the weighted average age at examination (43.6 years), proportion male (47.5%), proportion born before 1980 (80.0%), nativity/age at immigration categories, proportion for each background by study site combination (constructed to represent combinations of more than ≥100 individuals, see Table 1), and proportion with digit preference for self-reports ending in 0s or 5s (79.5%). **Panel B.** Among individuals who immigrated to the US first as adults (>21 years), the gray weight trajectory and 95% CI reflects the weighted average at examination (48.8 years), proportion male (45.3%), proportion immigrating before 1980 (8.4%), average age at immigration (35.5 years), proportion for each background by study site combination (see Table 1), and proportion with digit preference for self-reports ending in 0s or 5s (75.7%). The colored weight trajectories are based on the same model coefficients, but reflect the average adjustments and range of observed time points for the subset of participants who were either <45 years (blue) or ≥45 years (red) at baseline. The test of difference in pre/post immigration slope was significant (Chi-square p-value<0.0001). Examples of how to calculate population-level weight change, or the effect of demographic or sociocultural factors, based on the final model coefficients are provided as part of Supplemental Tables 3-4.

**Supplemental Table 1. Population-level effects included in linear mixed model for weight trajectories across age (Model 1)**

| **Main Effect^a^** | **Linear Term^a^** | **Quadratic Term^a^** |
| --- | --- | --- |
|  | Report age^b^ |  |
| Age at examination^c^ |  |  |
| Born before 1980 | (born before 1980) * (report age) | (born before 1980) * (report age)^2^ |
| Nativity/age at immigration categories | (nativity/age at immigration) * (report age) | (nativity/age at immigration) * (born before 1980) * (report age)^2^ |
| Male | (male) * (report age) | (male) * (born before 1980) * (report age)^2^ |
| Background-study site | (background-study site) * (report age) | (background-study site) * (born before 1980) * (report age)^2^ |
| End digit preference for 0s and 5s^d^ |  |  |
|  | **Final Equation** |  |
| Model 1: weight = report age + age at examination + born before 1980 + nativity/age at immigration categories + male + background-study site + end digit preference for 0s and 5s + (born before 1980)*(report age) + (born before 1980) *(report age)^2^ + (place of nativity/age at immigration)*(report age) + (place of nativity/age at immigration)*(born before 1980)*(report age)^2^ + (male)*(report age) + (male)*(born before 1980)*(report age)^2^ + (background-study site)*(report age), (background-study site)*(born before 1980)*(report age)^2^ + random intercept for primary sampling unit + random intercept for household + random intercept for individual + random individual-specific linear slope | | |

^a^ The “Main Effect” column lists main effects of covariates included in the model. The “Linear Term” column lists terms that are linear in report age (i.e., the time variable in the longitudinal model). The “Quadratic Term” column lists terms that are quadratic in report age.

^b^ Since participants born in 1980 or later had at most two weight measurements (one at age 25 and another at the baseline age), the weight trajectory was allowed to be quadratic for those born before 1980 and allowed to be linear for those born in 1980 or later. Therefore, all quadratic terms for report age were multiplied by “born before 1980” to ensure that the trajectories were only allowed to be quadratic among the subgroup that was born before 1980.

^c^ Age at examination was included to account for determinants of mis-reporting weight, and there was no interest in examining how the shape of the weight trajectories differed by the age at examination. Therefore, interactions between age at examination and report age (either linear or quadratic terms) were not included in the model.

^d^ End digit preference was included to account for determinants of mis-reporting weight, and there was no interest in examining how the shape of the weight trajectories differed by end digit preference. Therefore, interactions between end digit preference and report age (either linear or quadratic terms) were not included in the model.

**Supplemental Table 2. Population-level effects included in linear mixed model for weight trajectories across time since immigration (Model 2)**

|  | **Pre-Immigration^a^** | | **Post-Immigration^a^** | |
| --- | --- | --- | --- | --- |
| **Main Effect^a^** | **Linear Term** | **Quadratic Term** | **Linear Term** | **Quadratic Term** |
|  | Time since immigration | (Time since immigration)^2^ | (post-immigration) * (time since immigration) | (post-immigration) * (time since immigration)^2^ |
| Age at examination^b^ |  |  |  |  |
| Immigrated before 1980 | (immigrated before 1980) * (time since immigration) | (immigrated before 1980) * (time since immigration)^2^ | (immigrated before 1980) * (post-immigration) * (time since immigration) | (immigrated before 1980) * (post-immigration) * (time since immigration)^2^ |
| Age at immigration | (age at immigration) * (time since immigration) | (age at immigration) * (time since immigration)^2^ | (age at immigration) * (post-immigration) * (time since immigration) | (age at immigration) * (post-immigration) * (time since immigration)^2^ |
| Male | (male) * (time since immigration) | (male) * (time since immigration)^2^ | (male) * (post-immigration) * (time since immigration) | (male) * (post-immigration) * (time since immigration)^2^ |
| Background-study site | (background-study site) * (time since immigration) | (background-study site) * (time since immigration)^2^ | (background-study site) * (post-immigration) * (time since immigration) | (background-study site) * (post-immigration) * (time since immigration)^2^ |
| End digit preference for 0s and 5s^c^ |  |  |  |  |
|  |  | **Final Equation** |  |  |
| Model 2: time since immigration + time since immigration^2^ + (post-immigration)*(time since immigration) + (post-immigration)*(time since immigration)^2^ + age at examination + immigrated before 1980 + age at immigration + male + background-study site + end digit preference for 0s and 5s + (immigrated before 1980)*(time since immigration) + (immigrated before 1980)*(time since immigration)^2^ + (immigrated before 1980)*(post-immigration)*(time since immigration) + (immigrated before 1980)*(post-immigration)*(time since immigration)^2^ + (age at immigration)*(time since immigration) + (age at immigration)*(time since immigration)^2^ + (age at immigration)*(post-immigration)*(time since immigration) + (age at immigration)*(post-immigration)*(time since immigration)^2^ + (male)*(time since immigration) + (male)*(time since immigration)^2^ + (male)*(post-immigration)*(time since immigration) + (male)*(post-immigration)*(time since immigration)^2^ + (background-study site)*(time since immigration) + (background-study site)*(time since immigration)^2^ + (background-study site)*(post-immigration)*(time since immigration), (background-study site)*(post-immigration)*(time since immigration)^2^ + random intercept for primary sampling unit + random intercept for household + random intercept for individual + random individual-specific linear slope | | | | |

^a^ The “Main Effect” column lists main effects of covariates included in the model. The “Pre-immigration” columns list terms that are linear (“Linear Term” column) and quadratic (“Quadratic Term” column) in time since immigration (i.e., the time variable in the longitudinal model) for the pre-immigration part of the weight trajectories (i.e., for negative time since immigration). The “Post-immigration” columns list terms that are linear (“Linear Term” column) and quadratic (“Quadratic Term” column) in time since immigration for the post-immigration part of the weight trajectories (i.e., for positive time since immigration).

^b^ Age at examination was included to account for determinants of mis-reporting weight, and there was no interest in examining how the shape of the weight trajectories differed by the age at examination. Therefore, interactions between age at examination and report age (either linear or quadratic terms) were not included in the model.

^c^ End digit preference was included to account for determinants of mis-reporting weight, and there was no interest in examining how the shape of the weight trajectories differed by end digit preference. Therefore, interactions between end digit preference and report age (either linear or quadratic terms) were not included in the model.

**Supplemental Table 3. Population-level effects included in linear mixed model for weight trajectories across age (Model 1)**

| **Effect** | **Coefficient (SE)** |
| --- | --- |
| Intercept | 57.653 (1.135) |
| Report age ^†^ | 2.043 (0.116) |
| Centered age at examination ^‡^ | -0.334 (0.016) |
| Born before 1980 | 1.088 (0.608) |
| (born before 1980) * (report age) | -0.689 (0.069) |
| (born before 1980) * (report age)^2^ | -0.015 (0.003) |
| Nativity/age at immigration |  |
| Born in US | -- |
| < 12 years | -0.616 (1.145) |
| 12 – 21 years | -4.583 (0.674) |
| 22 – 34 years | -6.281 (0.641) |
| 35 – 44 years | -5.072 (0.635) |
| 45 – 74 years | -1.897 (0.711) |
| (nativity/age at immigration) * (report age) |  |
| Born in US | -- |
| < 12 years | -0.257 (0.099) |
| 12 – 21 years | -0.262 (0.070) |
| 22 – 34 years | -0.240 (0.068) |
| 35 – 44 years | -0.474 (0.067) |
| 45 – 74 years | -0.799 (0.075) |
| (nativity/age at immigration) * (born before 1980) * (report age)^2^ |  |
| Born in US | -- |
| < 12 years | 0.006 (0.003) |
| 12 – 21 years | 0.005 (0.002) |
| 22 – 34 years | 0.004 (0.002) |
| 35 – 44 years | 0.008 (0.002) |
| 45 – 74 years | 0.013 (0.002) |
| Male | 12.566 (0.330) |
| (male) * (report age) | -0.051 (0.029) |
| (male) * (born before 1980) * (report age)^2^ | 0.000 (0.001) |
| Background/study site |  |
| Bronx |  |
| Dominicans | -- |
| Central Americans | 0.298 (1.397) |
| Mexicans | -1.291 (0.944) |
| Puerto Ricans | 2.849 (0.799) |
| South Americans | 0.695 (1.267) |
| Chicago (all backgrounds) | 0.786 (0.721) |
| Miami |  |
| Central Americans | -0.370 (0.886) |
| Cubans | 1.163 (0.764) |
| South Americans | 2.670 (0.896) |
| San Diego |  |
| Mexicans | 3.718 (0.754) |
| Mixed/other backgrounds from Bronx, Miami, and San Diego | 2.808 (1.434) |
| (background/study site) * (report age) |  |
| Bronx |  |
| Dominicans | -- |
| Central Americans | -0.148 (0.103) |
| Mexicans | -0.062 (0.133) |
| Puerto Ricans | -0.275 (0.084) |
| South Americans | -0.359 (0.100) |
| Chicago (all backgrounds) | -0.149 (0.071) |
| Miami |  |
| Central Americans | 0.053 (0.077) |
| Cubans | -0.083 (0.073) |
| South Americans | -0.347 (0.095) |
| San Diego |  |
| Mexicans | -0.263 (0.072) |
| Mixed/other backgrounds from Bronx, Miami, and San Diego | -0.272 (0.101) |
| (background/study site) * (born before 1980) * (report age)^2^ |  |
| Bronx |  |
| Dominicans | -- |
| Central Americans | 0.004 (0.003) |
| Mexicans | -0.002 (0.004) |
| Puerto Ricans | 0.005 (0.002) |
| South Americans | 0.007 (0.003) |
| Chicago (all backgrounds) | 0.001 (0.002) |
| Miami |  |
| Central Americans | -0.002 (0.002) |
| Cubans | 0.002 (0.002) |
| South Americans | 0.006 (0.002) |
| San Diego |  |
| Mexicans | 0.004 (0.002) |
| Mixed/other backgrounds from Bronx, Miami, and San Diego | 0.005 (0.002) |
| End digit preference for 0s and 5s | -0.239 (0.186) |

^†^ Note that report age was centered at age 21 in the model. ^‡^ Note that age at examination was centered at age 48.69 years in the model.

Note that the slopes in Figures 1A, 2A, 2C, 2E, and 3A – 3D for a given age range and a given set of covariate values can be calculated based on the estimated regression coefficients reported in this table. For example, the slope from 22 – 30 years of age for a given set of covariate values can be calculated using the following formula: 2.043*(30 - 22) - .257*(age at immigration < 12 years)*(30 - 22) - .262*(age at immigration from 12 to 21 years)*(30 - 22) - .240*(age at immigration from 22 to 34 years)*(30 - 22) - .474*(age at immigration from 35 to 44 years)*(30 - 22) - .799*(age at immigration from 45 to 74 years)*(30 - 22) + .006*(age at immigration < 12 years)*(30^2^ – 22^2^)*(born before 1980) + .005*(age at immigration from 12 to 21 years)*(30^2^ – 22^2^)*(born before 1980) + .004*(age at immigration from 22 to 34 years)*(30^2^ – 22^2^)*(born before 1980) + .008*(age at immigration from 35 to 44 years)*(30^2^ – 22^2^)*(born before 1980) + .013*(age at immigration from 45 - 74 years)*(30^2^ – 22^2^)*(born before 1980) - .148*(Central Americans from Bronx)*(30 - 22) - .149*(any Hispanic background from Chicago)*(30 - 22) + .053*(Central Americans from Miami)*(30 - 22) - .083*(Cubans from Miami)*(30 - 22) - .062*(Mexicans from Bronx)*(30 - 22) - .263*(Mexicans from San Diego)*(30 - 22) - .275*(Puerto Ricans from Bronx)*(30 - 22) - .359*(South Americans from Bronx)*(30 - 22) - .347*(South Americans from Miami)*(30 - 22) - .272*(mixed/other backgrounds from Bronx, Miami, and San Diego)*(30 - 22) + .004*(Central Americans from Bronx)*(30^2^ – 22^2^)*(born before 1980) + .001*(any Hispanic background from Chicago)*( 30^2^ – 22^2^)*(born before 1980) - .002*(Central Americans from Miami)*( 30^2^ – 22^2^)*(born before 1980) + .002*(Cubans from Miami)*( 30^2^ – 22^2^)*(born before 1980) - .002*(Mexicans from Bronx)*( 30^2^ – 22^2^)*(born before 1980) + .004*(Mexicans from San Diego)*( 30^2^ – 22^2^)*(born before 1980) + .005*(Puerto Ricans from Bronx)*( 30^2^ – 22^2^)*(born before 1980) + .007*(South Americans from Bronx)*( 30^2^ – 22^2^)*(born before 1980) + .006*(South Americans from Miami)*( 30^2^ – 22^2^)*(born before 1980) + .005*(mixed/other backgrounds from Bronx, Miami, and San Diego)*( 30^2^ – 22^2^)*(born before 1980) - .051*(30 - 22)*(male) + .000*(30^2^ – 22^2^)*(male)*(born before 1980) - .689*(30 - 22)*(born before 1980) - .015*(30^2^ – 22^2^)*(born before 1980).

**Supplemental Table 4. Population-level effects included in linear mixed model for weight trajectories across time since immigration (Model 2)**

| **Effect** | **Coefficient (SE)** |
| --- | --- |
| Intercept | 45.894 (1.308) |
| Time since immigration | 0.627 (0.272) |
| (time since immigration)^2^ | -0.002 (0.014) |
| (post-immigration) * (time since immigration) | 0.492 (0.377) |
| (post-immigration) * (time since immigration)^2^ | -0.000 (0.014) |
| Age at examination ^†^ | -0.324 (0.021) |
| Immigrated after 1980 | -1.227 (1.027) |
| (immigrated after 1980) * (time since immigration) | 0.611 (0.253) |
| (immigrated after 1980) * (time since immigration)^2^ | 0.019 (0.014) |
| (immigrated after 1980) * (post-immigration) * (time since immigration) | -0.069 (0.317) |
| (immigrated after 1980) * (post-immigration) * (time since immigration)^2^ | -0.035 (0.013) |
| Age at immigration | 0.482 (0.024) |
| (age at immigration) * (time since immigration) | -0.015 (0.003) |
| (age at immigration) * (time since immigration)^2^ | -0.000 (0.000) |
| (age at immigration) * (post-immigration) * (time since immigration) | -0.002 (0.005) |
| (age at immigration) * (post-immigration) * (time since immigration)^2^ | 0.000 (0.000) |
| Male | 11.621 (0.472) |
| (male) * (time since immigration) | 0.123 (0.052) |
| (male) * (time since immigration)^2^ | 0.004 (0.001) |
| (male) * (post-immigration) * (time since immigration) | -0.264 (0.095) |
| (male) * (post-immigration) * (time since immigration)^2^ | -0.001 (0.002) |
| Background/study site |  |
| Bronx |  |
| Dominicans | -- |
| Central Americans | 0.417 (1.541) |
| Mexicans | -0.605 (2.033) |
| Puerto Ricans | 4.659 (1.297) |
| South Americans | -1.966 (1.484) |
| Chicago (all backgrounds) | -0.220 (0.909) |
| Miami |  |
| Central Americans | -0.824 (1.004) |
| Cubans | 1.292 (0.905) |
| South Americans | 0.771 (1.491) |
| San Diego |  |
| Mexicans | 3.023 (0.912) |
| Mixed/other backgrounds from Bronx, Miami, and San Diego | 2.111 (1.539) |
| (background/study site) * (time since immigration) |  |
| Bronx |  |
| Dominicans | -- |
| Central Americans | -0.143 (0.197) |
| Mexicans | 0.426 (0.322) |
| Puerto Ricans | 0.328 (0.164) |
| South Americans | -0.386 (0.192) |
| Chicago (all backgrounds) | -0.020 (0.110) |
| Miami |  |
| Central Americans | -0.124 (0.116) |
| Cubans | 0.080 (0.102) |
| South Americans | -0.270 (0.162) |
| San Diego |  |
| Mexicans | -0.054 (0.116) |
| Mixed/other backgrounds from Bronx, Miami, and San Diego | 0.220 (0.171) |
| (background/study site) * (time since immigration)^2^ |  |
| Bronx |  |
| Dominicans | -- |
| Central Americans | -0.002 (0.006) |
| Mexicans | 0.018 (0.010) |
| Puerto Ricans | 0.010 (0.006) |
| South Americans | -0.013 (0.005) |
| Chicago (all backgrounds) | 0.001 (0.003) |
| Miami |  |
| Central Americans | -0.003 (0.003) |
| Cubans | 0.001 (0.003) |
| South Americans | -0.005 (0.005) |
| San Diego |  |
| Mexicans | -0.001 (0.003) |
| Mixed/other backgrounds from Bronx, Miami, and San Diego | 0.006 (0.004) |
| (background/study site) * (post-immigration) * (time since immigration) |  |
| Bronx |  |
| Dominicans | -- |
| Central Americans | 0.022 (0.341) |
| Mexicans | -0.499 (0.580) |
| Puerto Ricans | -0.384 (0.267) |
| South Americans | 0.355 (0.336) |
| Chicago (all backgrounds) | -0.002 (0.204) |
| Miami |  |
| Central Americans | 0.255 (0.229) |
| Cubans | 0.133 (0.200) |
| South Americans | 0.195 (0.267) |
| San Diego |  |
| Mexicans | -0.138 (0.203) |
| Mixed/other backgrounds from Bronx, Miami, and San Diego | -0.556 (0.309) |
| (background/study site) * (post-immigration) * (time since immigration)^2^ |  |
| Bronx |  |
| Dominicans | -- |
| Central Americans | 0.006 (0.007) |
| Mexicans | -0.030 (0.012) |
| Puerto Ricans | -0.013 (0.006) |
| South Americans | 0.014 (0.006) |
| Chicago (all backgrounds) | -0.004 (0.003) |
| Miami |  |
| Central Americans | -0.002 (0.005) |
| Cubans | -0.008 (0.004) |
| South Americans | 0.005 (0.006) |
| San Diego |  |
| Mexicans | 0.001 (0.004) |
| Mixed/other backgrounds from Bronx, Miami, and San Diego | 0.002 (0.005) |
| End digit preference for 0s and 5s | -0.154 (0.207) |

^†^ Note that age at examination was centered at age 48.69 years in the model.

Note that the slopes in Figures 1B, 2B, 2D, 2F, and 3E – 3H for a given period of time and a given set of covariate values can be calculated based on the estimated regression coefficients reported in this table. For example, the slope for the 5 years pre-migration for a given set of covariate values can be calculated using the following formula: .627*(0 – (-5)) - .002*(0^2^ – (-5)^2^) + .123*(0 – (-5))*(male) + .004*(0^2^ – (-5)^2^)*(male) - .143*(0 – (-5))*(Central Americans from Bronx) - .020*(0 – (-5))*(any Hispanic background from Chicago) - .124*(0 – (-5))*(Central Americans from Miami) + .080*(0 – (-5))*(Cubans from Miami) + .426*(0 – (-5))*(Mexicans from Bronx) - .054*(0 – (-5))*(Mexicans from San Diego) + .328*(0 – (-5))*(Puerto Ricans from Bronx) - .386*(0 – (-5))*(South Americans from Bronx) - .270*(0 – (-5))*(South Americans from Miami) + .220*(0 – (-5))*(mixed/other backgrounds from Bronx, Miami, and San Diego) - .002*(0^2^ – (-5)^2^)*(Central Americans from Bronx) + .001*(0^2^ – (-5)^2^)*(any Hispanic background from Chicago) - .003*(0^2^ – (-5)^2^)*(Central Americans from Miami) + .001*(0^2^ – (-5)^2^)*(Cubans from Miami) + .018*(0^2^ – (-5)^2^)*(Mexicans from Bronx) - .001*(0^2^ – (-5)^2^)*(Mexicans from San Diego) + .010*(0^2^ – (-5)^2^)*(Puerto Ricans from Bronx) - .013*(0^2^ – (-5)^2^)*(South Americans from Bronx) - .005*(0^2^ – (-5)^2^)*(South Americans from Miami) + .006*(0^2^ – (-5)^2^)*(mixed/other backgrounds from Bronx, Miami, and San Diego) - .015*(0 – (-5))*(age at immigration) - .000*(0^2^ – (-5)^2^)*(age at immigration) + .611*(0 – (-5))*(immigrated after 1980) + .019*(0^2^ – (-5)^2^)*(immigrated after 1980)

For example, the slope for the 5 years post-migration for a given set of covariate values can be calculated using the following formula: .627*(5 – 0) - .002*(5^2^ – 0^2^) + .492*1*(5 – 0) - .000*1*(5^2^ – 0^2^) + .123*(5 – 0)*(male) + .004*(5^2^ – 0^2^)*(male) - .264*1*(5 – 0)*(male) - .001*1*(5^2^ – 0^2^)*(male) - .143*(5 – 0)*(Central Americans from Bronx) - .020*(5 – 0)*(any Hispanic background from Chicago) - .124*(5 – 0)*(Central Americans from Miami) + .080*(5 – 0)*(Cubans from Miami) + .426*(5 – 0)*(Mexicans from Bronx) - .054*(5 – 0)*(Mexicans from San Diego) + .328*(5 – 0)*(Puerto Ricans from Bronx) - .386*(5 – 0)*(South Americans from Bronx) - .270*(5 – 0)*(South Americans from Miami) + .220*(5 – 0)*(mixed/other backgrounds from Bronx, Miami, and San Diego) - .002*(5^2^ – 0^2^)*(Central Americans from Bronx) + .001*(5^2^ – 0^2^)*(any Hispanic background from Chicago) - .003*(5^2^ – 0^2^)*(Central Americans from Miami) + .001*(5^2^ – 0^2^)*(Cubans from Miami) + .018*(5^2^ – 0^2^)*(Mexicans from Bronx) - .001*(5^2^ – 0^2^)*(Mexicans from San Diego) + .010*(5^2^ – 0^2^)*(Puerto Ricans from Bronx) - .013*(5^2^ – 0^2^)*(South Americans from Bronx) - .005*(5^2^ – 0^2^)*(South Americans from Miami) + .006*(5^2^ – 0^2^)*(mixed/other backgrounds from Bronx, Miami, and San Diego) + .022*1*(5 – 0)*(Central Americans from Bronx) - .002*1*(5 – 0)*(any Hispanic background from Chicago) + .255*1*(5 – 0)*(Central Americans from Miami) + .133*1*(5 – 0)*(Cubans from Miami) - .499*1*(5 – 0)*(Mexicans from Bronx) - .138*1*(5 – 0)*(Mexicans from San Diego) - .384*1*(5 – 0)*(Puerto Ricans from Bronx) + .355*1*(5 – 0)*(South Americans from Bronx) + .195*1*(5 – 0)*(South Americans from Miami) - .556*1*(5 – 0)*(mixed/other backgrounds from Bronx, Miami, and San Diego) + .006*1*(5^2^ – 0^2^)*(Central Americans from Bronx) - .004*1*(5^2^ – 0^2^)*(any Hispanic background from Chicago) - .002*1*(5^2^ – 0^2^)*(Central Americans from Miami) - .008*1*(5^2^ – 0^2^)*(Cubans from Miami) - .030*1*(5^2^ – 0^2^)*(Mexicans from Bronx) + .001*1*(5^2^ – 0^2^)*(Mexicans from San Diego) - .013*1*(5^2^ – 0^2^)*(Puerto Ricans from Bronx) + .014*1*(5^2^ – 0^2^)*(South Americans from Bronx) + .005*1*(5^2^ – 0^2^)*(South Americans from Miami) + .002*1*(5^2^ – 0^2^)*(mixed/other backgrounds from Bronx, Miami, and San Diego) - .015*(5 – 0)*(age at immigration) - .000*(5^2^ – 0^2^)*(age at immigration) - .002*1*(5 – 0)*(age at immigration) + .000*1*(5^2^ – 0^2^)*(age at immigration) + .611*(5 – 0)*(immigrated after 1980) + .019*(5^2^ – 0^2^)*(immigrated after 1980) - .069*1*(5 – 0)*(immigrated after 1980) - .035*1*(5^2^ – 0^2^)*(immigrated after 1980)

**Supplemental Table 5. Weight mean heights and estimated weights corresponding to a body mass index (BMI) of 30kg/m^2^ for the analytic subsamples included in the linear mixed model for weight trajectories across age (Model 1) and time since immigration (Model 2) overall and across strata of key demographic and sociocultural factors**

|  |  | *Overall Model 1*  *(Unweighted n=15,203)* | | *Adult Immigrant Subpopulation^‡^ Model 2*  *(Unweighted n=8,830)* | |
| --- | --- | --- | --- | --- | --- |
|  |  | **Height (cm)** | **First Obese Weight (kg)** ^†^ | **Height (cm)** | **First Obese Weight (kg)** ^†^ |
| ***Overall*** | *All ages* | 163.37 | 80.07 | 162.21 | 78.94 |
|  | *<45 years* | 164.44 | 81.12 | 163.23 | 79.93 |
|  | *45+ years* | 162.06 | 78.79 | 161.51 | 78.26 |
| ***Birth or Immigration Cohort*** | *Before 1980* | 162.93 | 79.64 | 160.68 | 77.45 |
|  | *1980 or after* | 165.10 | 81.77 | 162.35 | 79.07 |
| ***Gender*** | *Female* | 157.04 | 73.98 | 156.22 | 73.21 |
|  | *Male* | 170.36 | 87.07 | 169.42 | 86.11 |
| ***Nativity and***  ***Age at Immigration (years)*** | *US-born* | 166.64 | 83.31 | - | - |
|  | *Birth to 11* | 163.88 | 80.57 |  |  |
|  | *12 to 21* | 163.12 | 79.82 |  |  |
|  | *22 to 34* | 162.56 | 79.28 | 162.56 | 79.28 |
|  | *35 to 44* | 162.51 | 79.23 | 162.51 | 79.23 |
|  | *45 to 54* | 160.90 | 77.67 | 161.72 | 78.46 |
|  | *55 to 64* |  |  | 159.74 | 76.55 |
|  | *65+* |  |  | 159.55 | 76.37 |
| ***Background by Study Site***  *Bronx, NY* | *Dominican* | 163.26 | 79.96 | 162.17 | 78.90 |
|  | *Central Am.* | 162.70 | 79.41 | 160.37 | 77.16 |
|  | *Mexican* | 158.02 | 74.91 | 156.49 | 73.47 |
|  | *Puerto Rican* | 163.90 | 80.59 | 162.49 | 79.21 |
|  | *South Am.* | 161.78 | 78.52 | 160.45 | 77.23 |
| *Chicago, IL* | *Combined* | 162.19 | 78.92 | 159.99 | 76.79 |
| *Miami, FL* | *Central Am.* | 160.84 | 77.61 | 159.73 | 76.54 |
|  | *Cuban* | 164.69 | 81.37 | 164.20 | 80.88 |
|  | *South Am.* | 162.89 | 79.60 | 162.34 | 79.06 |
| *San Diego, CA* | *Mexican* | 163.52 | 80.22 | 162.18 | 78.91 |
| ***Non-Obese at 21 Years*^⸸^** | *All ages* | 163.26 | 79.96 | 162.19 | 78.92 |
|  | *<45 years* | 164.24 | 80.92 | 163.06 | 79.77 |
|  | *45+ years* | 162.15 | 78.88 | 161.61 | 78.35 |

Height values were weighted for study design and non-response. Am.=American

^†^ Note that the ‘First Obese Weight (kg)’ for an average participant in a given subsample and/or stratum corresponds a BMI of 30kg/m^2^. This value was calculated using the weighted mean height in cm and the following formula: ((height in cm)/100)^2^ *30.

^‡^ Note that Model 2 includes only adults who migrated to the 50 US states and DC at >21 years old.

^⸸^ Note that the subset not living with obesity (body mass index<30 kg/m^2^) at 21 years included 13,125 of the overall analytic sample and 7,763 of the adult immigrant subpopulation.
